# Spontaneous splenic hematoma secondary to dengue infection: a rare case report

**DOI:** 10.1097/MS9.0000000000000312

**Published:** 2023-03-16

**Authors:** Soumya Pahari, Sunil Basukala, Prakash Kunwar, Kriti Thapa, Yugant Khand, Ojas Thapa

**Affiliations:** a Nepalese Army Institute of Health Sciences (NAIHS), Sanobharyang; b Department of Surgery, Shree Birendra Hospital, Chhauni, Kathmandu, Nepal

**Keywords:** case report, dengue hemorrhagic fever, dengue, shock, splenic hematoma

## Abstract

Dengue infection may have various surgical complications. Splenic hematoma is a rare complication of dengue hemorrhagic fever and may be life-threatening.

**Case Presentation:**

A 54-year-old male, diagnosed with dengue infection detected at another hospital, presented on the 10th day of fever with left upper abdominal pain for 7 days without history of trauma. Urgent ultrasonography of the abdomen revealed findings suggestive of a splenic subcapsular hematoma, which was confirmed by computed tomography scan. The grade II splenic hematoma was being managed conservatively. Unfortunately, the patient developed hospital acquired pneumonia and died from septic shock.

**Clinical Discussion:**

Hemorrhagic manifestations are seen in the febrile and critical phase of dengue, but the spleen is infrequently involved. Splenic hematoma can lead to splenic rupture, which can be rapidly fatal. Specific treatment guidelines of such hematomas are needed in the context of dengue infection, as the treatment modality is controversial.

**Conclusion:**

Patients must be carefully evaluated for the complications and surgical manifestations of dengue as abdominal pain and hypotension from splenic hematoma may be misinterpreted as components of dengue hemorrhagic fever and dengue shock syndrome.

## Introduction

Highlights

- Hemorrhagic manifestations occur in dengue infection, but splenic hematoma is rare.
- It may lead to splenic rupture, which can be fatal.
- Surgical complications must be kept in mind when dealing with abdominal pain in dengue infection.
- Splenic hematoma and rupture may mimic dengue shock syndrome, hence may be missed.

Dengue is a febrile illness caused by infection with dengue virus, which is transmitted by the vector: female *Aedes* mosquito. The clinical presentation following a dengue infection has a wide spectrum from being asymptomatic or a mild febrile illness only to severe life-threatening hemorrhagic manifestations and shock syndrome^1^–^3^.

It has three phases of infection: the febrile phase, critical phase, and the convalescent (recovery) phase^2^. Most patients recover from the febrile phase without complications. Some patients who progress into the critical phase develop plasma leakage, bleeding, shock, and organ impairment^2^. This leads to complications varying from encephalopathy, pulmonary effusion, ascites, Acute Kidney Injury, internal hemorrhage to Disseminated Intravascular Coagulopathy. WHO introduced the concept of ‘Expanded Dengue Syndrome’ for the unusual/atypical manifestations of dengue, which include neurologic, cardiac, hepatic, renal, and other isolated organ involvement including the spleen^3^. Dengue infection may have surgical manifestations, mainly acute cholecystitis, acute pancreatitis, acute appendicitis, splenic rupture, bowel perforation, gastrointestinal bleeding, and hematomas^4^. Splenic hematoma in dengue hemorrhagic fever (DHF) is rare and present in 1.5% of cases^5^. Nepal being one of the endemic countries for dengue and in the current setting of a nationwide epidemic as of October 2022, we encountered this rare complication of dengue as splenic hematoma in a patient with dengue fever as discussed below. This case report has been reported in line with the SCARE Criteria^6^.

## Case presentation

**Figure 1:**
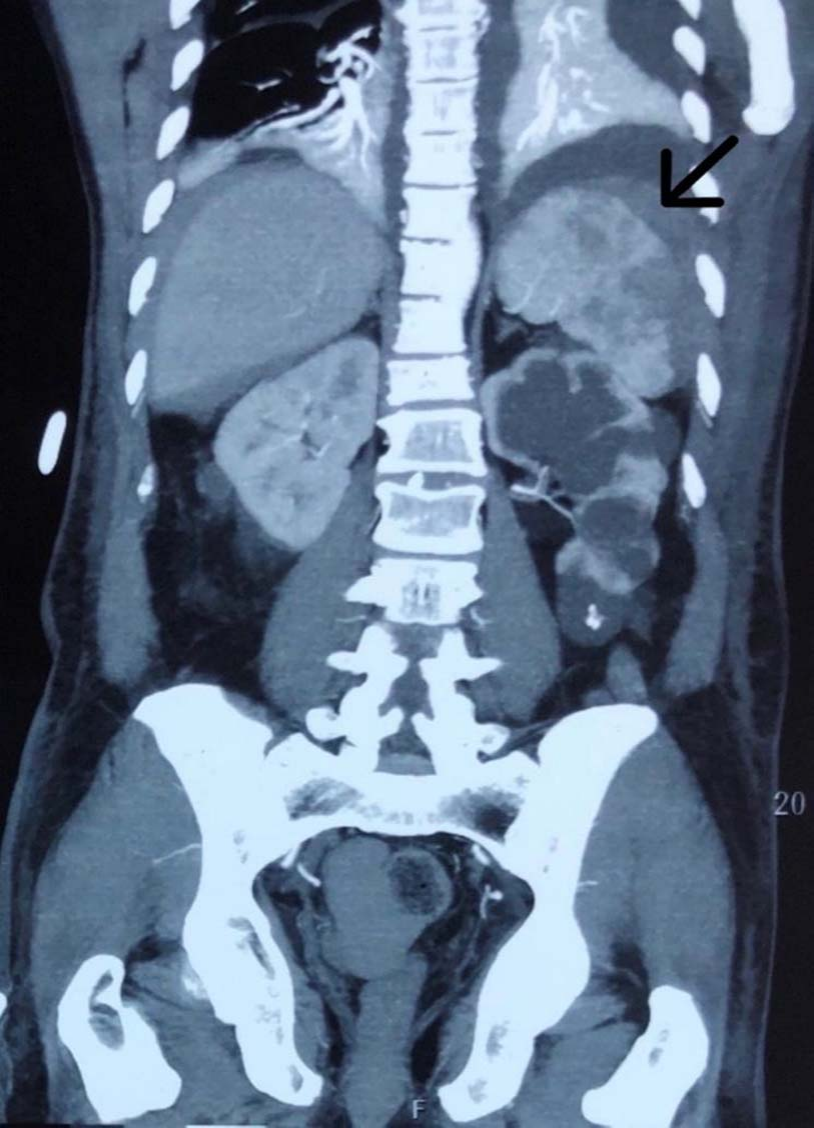
Contrast enhanced computed tomography scan of abdomen (coronal view) showing grade II splenic hematoma.

A 57-year-old male from rural Nepal presented to the emergency department with pain on the left upper abdomen for 1 week. He was diagnosed with dengue infection (NS1 Ag positive) following a febrile illness 10 days back at his local hospital. The pain was continuous, dull aching in nature, nonradiating, relieved partially by analgesics, and moderate in severity. His past medical history, personal history, family history, and allergic history were unremarkable. On examination, he was ill-looking with vitals within the normal limits. There was a marked tenderness over the left hypochondrium. Other systemic examinations were noncontributory. Initial investigations revealed, hemoglobin 12.3 g/dl, total leukocyte count 8.3×10^3^/µl (N=70%, L=22%), platelet count 60 000/µl. Renal function test showed value of urea 70 mg/dl, creatinine 1.6 mg/dl, sodium 132 mEq/l, potassium 3.4 mEq/l. Serum amylase, total serum bilirubin, and direct serum bilirubin, and coagulation profile and liver enzymes were within normal limits. Dengue profile revealed serum NS1 Ag and immunoglobulin M antibody positive. Urgent Ultrasound Guidance of the abdomen and pelvis revealed splenomegaly with splenic subcapsular collections with low-level echogenicity within measuring 4.1×4.8×2.1 cm/22 ml suggestive of splenic subcapsular hematoma. He was resuscitated with intravenous fluid. Two pints of platelet rich plasma were transfused in line with his low platelet count and evidence of hemorrhage. Contrast enhanced computed tomography scan of the abdomen was done, which confirmed grade II splenic hematoma (Fig. 1) which was planned to be managed conservatively. On the third day of admission, there was a drop in his blood pressure requiring inotropic support. He was intubated for deteriorating consciousness and mechanical ventilation was started. His lab parameters showed rising leukocyte count 16.5×10^3^/µl (N=81%, L=16%). Sepsis with septic shock was considered and broad spectrum antibiotics were started. On the fifth day of admission, his chest radiography findings showed patchy consolidations over the right lung field suggestive of hospital acquired pneumonia. He remained hypotensive despite maximal inotropic support and fluid resuscitation. A bed-side ultrasonography of the abdomen was considered in the view of a possible splenic rupture. However, it showed no evidence of hematoma rupture or free intraperitoneal fluid. Since his vitals were unstable, a repeat computed tomography scan of the abdomen could not be performed. He subsequently passed away on the fifth day of admission due to refractory septic shock.

## Discussion

Clinically, a patient with dengue infection usually presents with high-grade fever with chills and rigors associated with headache, myalgia, arthralgia, and ocular pain. Other symptoms include rashes, abdominal pain, vomiting, mucosal bleeding, and purpura along with laboratory findings of decreased platelet count <150 000/mm^3^, raised hematocrit and elevated liver enzymes^1^. The risk of infection and severity of disease is thought to be impacted by multiple factors, each of the virus, the vector, and the host. Severe dengue has emerged as a major problem due to the hyperendemic transmission of multiple dengue viruses 1–4 from many geographical areas. Approximately 500 000 cases of severe dengue occur annually with a mortality rate of around 2.5%^1^.

The hemorrhagic manifestation can occur in any of the febrile or critical phase of infection. The induction of vascular permeability and shock depends on multiple factors, such as the presence or absence of enhancing and non-neutralizing antibodies, age, sex, race, nutritional status, and the sequence of infections^1^. Susceptibility to severe dengue drops considerably after 12 years of age. However, in recent years, the involvement of the adult population has been increasing due to a shifting pattern of immunity and the geographical spread of dengue illness^7^. The term ‘Expanded Dengue Syndrome’ has been introduced to describe the atypical presentation of dengue infection involving different organs, including the lymphoreticular system^3^. Furthermore, a myriad of tropical diseases like scrub typhus, amebiasis, malaria, etc., need to be considered in a patient originating from tropical disease endemic areas and presenting with abdominal pain, as it can mimic common conditions encountered in the west^8^,^9^.

Splenic involvement, which is infrequently observed in dengue infection, included splenic enlargement, splenic rupture, splenic infarction, and splenic hematoma. Splenic enlargement may be underreported and is present in 1–15% cases. Spontaneous splenic rupture is rare and might be fatal. Subcapsular hemorrhage aggravated by vascular abnormalities and thrombocytopenia is thought to lead to splenic rupture^5^,^7^,^10^,^11^.

Subcapsular splenic hematomas are usually associated with abdominal traumas. Nontraumatic causes include coagulopathies, infectious mononucleosis and rarely, pancreatitis, and cocaine use. About 1.5% of cases with DHF may have such a complication^5^,^12^. Most splenic complications reported in the literature are manifested in the acute phases of the illness and those occurring in the recovery phase have been sparsely reported^13^. The exact mechanism of splenic hematoma is unknown, but is not limited to thrombocytopenia. Coagulation factor deficiency from dengue infection, invasion of splenic endothelium from the virus, and liver damage are postulated to play a role^5^,^14^. Though our patient was in the recovery phase during the presentation, the hematoma likely occurred during the acute phase of illness, given the history of onset of left-sided abdominal pain from the fourth day of fever. Splenic hematoma might lead to splenic rupture, which can be rapidly fatal, unless quickly diagnosed and managed appropriately in time. Its diagnosis can be easily missed, as the hypotension from the splenic rupture may be misinterpreted as dengue shock syndrome. Abdominal imaging must be considered in a dengue infection presenting with hypotension and abdominal pain, keeping the possibility of splenic rupture in mind^15^,^16^.

In our case, since the splenic hematoma was of grade II according to the American Association for the Surgery of Trauma splenic injury scale, it was managed conservatively.

The treatment of subcapsular hematoma in DHF is controversial. Some authors advise early aggressive treatment with splenectomy to prevent splenic rupture, which can be fatal. Whereas conservative management has also been described, avoiding the mortality and morbidity associated with splenectomy. In general, splenic hematomas are most commonly traumatic, and 50–70% can be managed nonoperatively and resolve spontaneously in 2–3 months. However, given the bleeding tendency and multiple organ involvement in DHF, definitive guidelines should be established. Dronamraju, *et al*.^5^ reported ultrasonography-guided percutaneous drainage of subcapsular splenic hematoma and splenic artery embolization as a safe and effective option to prevent splenic rupture in a patient with DHF^17^.

This case report also highlights that though abdominal pain is common in dengue infection, it must be carefully evaluated keeping the surgical manifestations and complications of dengue infection in mind.

## Conclusion

Patients must be carefully evaluated for the complications and surgical manifestations of dengue, as abdominal pain and hypotension from splenic hematoma and splenic rupture may be misinterpreted as components of DHF and dengue shock syndrome.

## Ethical approval

The case report is exempt from ethical approval in our institution.

## Consent

Written informed consent was obtained from the patient for the publication of this case report and accompanying images. A copy of the written consent is available for review by the Editor-in-Chief of this journal on request.

## Sources of funding

This research did not receive any specific grant from funding agencies in the public, commercial, or not-for-profit sectors.

## Author contribution

S.B.: conceptualization and supervision. S.P., P.K., K.T.: writing – original draft. S.P., P.K., K.T., S.B., Y.K., and O.T.: writing – review and editing. All the authors read and approved the final manuscript.

## Conflicts of interest disclosure

All authors declare that they have no conflict of interest.

## Research registration unique identifying number (UIN)

1. Name of the registry: not applicable.
2. Unique identifying number or registration ID: not applicable.
3. Hyperlink to your specific registration (must be publicly accessible and will be checked): not applicable.

## Guarantor

Sunil Basukala.

## Provenance and peer review

Not commissioned, externally peer-reviewed.
